# Recognition of musical beat and style and applications in interactive humanoid robot

**DOI:** 10.3389/fnbot.2022.875058

**Published:** 2022-08-04

**Authors:** Yue Chu

**Affiliations:** Music College, Dalian University, Dalian, China

**Keywords:** multi-modal features, humanoid robot, recurrent neural network, recognition technologies, musical beat and recognition of style source

## Abstract

The musical beat and style recognition have high application value in music information retrieval. However, the traditional methods mostly use a convolutional neural network (CNN) as the backbone and have poor performance. Accordingly, the present work chooses a recurrent neural network (RNN) in deep learning (DL) to identify musical beats and styles. The proposed model is applied to an interactive humanoid robot. First, DL-based musical beat and style recognition technologies are studied. On this basis, a note beat recognition method combining attention mechanism (AM) and independent RNN (IndRNN) [AM-IndRNN] is proposed. The AM-IndRNN can effectively avoid gradient vanishing and gradient exploding. Second, the audio music files are divided into multiple styles using the music signal's temporal features. A human dancing robot using a multimodal drive is constructed. Finally, the proposed method is tested. The results show that the proposed AM-IndRNN outperforms multiple parallel long short-term memory (LSTM) models and IndRNN in recognition accuracy (88.9%) and loss rate (0.0748). Therefore, the AM-optimized LSTM model has gained a higher recognition accuracy. The research results provide specific ideas for applying DL technology in musical beat and style recognition.

## Introduction

Music is an indispensable part of modern life, which can assist the expression of emotion in different situations. Musical factors are complex. A composer considers some basic elements in his/her music composition: rhythm, melody, harmony, and timbre. Thus, one must gain a professional understanding of the basic musical elements to understand musical contents or themes. Non-professionals can also empathize with music through musical styles and beats. Of these, style is the overall grasp of music and people's intuitive feeling of a piece of music. Most music-playing software recommends music through users' historical musical style selections. Music recommendation accuracy has become the key metric for users in choosing music recommendation applications (APPs). The beat in music is generally understood as the combination law between strong and weak rhythms, reflected in a stressed note in a piece of a song. Following the musical beat, people will swing their bodies unconsciously or perform other activities. The recognition of style and beat is of great significance to robot performance (Abbaspour et al., 2020; Zhou et al., 2020). People can obtain massive amounts of audio, image, and video information through the internet. Internet music has a large user base, and the internet music library is enriched with the diversification of user needs. Given the massive amount of music information, users urgently demand a personalized information retrieval approach. However, music recommendation is extremely challenging. As a form of artistic expression, music is endowed with a certain emotion. It involves trivial elements, such as melody, rhythm, harmony, and form, thus forming different musical styles. Usually, different music styles emphasize different music elements. These features in music styles can be used in music classification and retrieval through the content information. Currently, the music style classification is most commonly studied and has seen successful commercialization by music dealers to organize and describe music. On the other hand, with the increased capacity of the Internet music library, style-based music retrieval has become the mainstream method of music information retrieval. Classifying music by style can meet the users' personalized music retrieval and facilitate users to retrieve and efficiently manage their preference music styles timely. At the same time, it is convenient for music dealers to manage and label music styles and recommend music styles of interest to users. Automatic and accurate classification and recognition of music styles can effectively reduce labor costs. Therefore, improving the accuracy of music style classification and recognition can promote the intelligent development of music platforms. It provides better services for music listeners, improves user experience, and expands their choices, which have great research and economic value.

As artificial intelligence (AI) research becomes mature, its application gets closer to public life. For example, intelligent robots are seeing various applicational scenarios, such as service robots and unmanned aerial vehicles (UAVs). Meanwhile, robotic technologies are oriented toward entertainment from practical works. Research on service-oriented robots is abundant both in and outside China, while there is relatively little research on dancing robots. Dancing to the beat might seem natural to a human, but getting robots to respond to beats requires tons of work and design.

Chronologically, Robots' applications can be segmented into several phases, from industrial robots to service robots and household robots. From the economic sector's perspective, robot applications are experienced practical->industrial-entertainment->domestic development stage. Researchers have also done many works in robotics, deep learning (DL), and music interaction in robotics. Wen (2020) designed an intelligent background music system based on DL, the internet of things (IoT), and the support vector machine (SVM). They used a recurrent neural network (RNN) structure to extract image features. Nam et al. (2019) developed an automatic string plucking system for guitar robots to generate music without machine noise. The soft robot technology was used for a new silent actuator: a soft elastic cone as a buffer to prevent impact noise. As a result, an elastic cone design method based on nonlinear finite element analysis (FEA) was proposed. The silent characteristics of the silent actuator were confirmed by the noise test that compares the silent actuator with the traditional actuator. Rajesh and Nalini (2020) represented that music was an effective medium to convey emotions. Emotional recognition in music was the process of recognizing emotions from music fragments. They proposed an instrument-like emotional recognition method in view of DL technology. The music data set was collected from strung, percussion, woodwind, and brass instruments corresponding to four emotions, namely, happiness, sadness, neutrality, and fear. From the instrumental data set, the features of Mel frequency cepstral coefficient (MFCC), normalization statistics of chroma energy, short-term Fourier Transform (FT) of chroma, spectral characteristic, spectral centroid, bandwidth, attenuation, and time characteristics were extracted. Based on the extracted features, the RNN was trained for emotional recognition. Then, the performance of RNN and baseline machine learning (ML) classification algorithm was compared. The results showed that deep RNN had an excellent effect on instrument emotional recognition. Instrument classes played an important role in music-induced emotions. Briot and Pachet (2020) indicated that in addition to traditional tasks, such as prediction, classification, and translation, DL was receiving increasing interest as a music generation method. The latest research groups, such as Google's Magenta and Spotify's Creator Technology Research Lab (CTRL), were evidenced. The motivation was to automatically use DL architecture to learn music style from any music corpus and then generate samples from the estimated distribution. Then, DL-based music generation reached certain limitations, such as feedforward in circular architecture, because they tended to imitate the learned corpus without the incentive of creativity. Besides, the DL architecture did not provide a direct method to control music generation. DL architecture automatically generated music without human-computer interaction (HCI). However, given its generated content, it still could not help musicians create and refine music. They focused on the issues of control, creativity, and interaction analysis. Then the limitations of applying DL to music generation were listed, and possible solutions were outlooked. Martin-Gutierrez et al. (2020) pointed out that the application of multimedia promoted the services provided by platforms, such as Spotify, Lastfm, or Billboard. However, the innovative methods of retrieving specific information from a large amount of music-related data have become a potential challenge in music information retrieval. They studied the creation of SpotGenTrack popular data sets. They proposed an innovative multi-mode end-to-end DL architecture HitMusicNet to predict the popularity of music recording. Experiments showed that the architecture proposed was better than the existing technology.

The innovation of this work is to propose a lightweight multi-task cascaded convolutional neural network (MTCNN). With the help of the proposed MTCNN, the notes are located and extracted for normalization operation. The innovative combination of independent recurrent neural network (RNN) and attention mechanism (AM) is used for music style recognition, and the data are transferred to the multi-attention CNN and long-short term memory (LSTM) network for feature extraction and recognition. This method does not need to label in advance and is weak-supervised learning. The refined features extracted by the multi-attention CNN increase the sample details and contain the global information of the samples. The method proposed improves the accuracy and precision of music style recognition to a certain extent.

## Design of music style recognition model and construction of interactive robot system

### Music style recognition modeling by IndRNN

The music style describes the overall characteristics of a complete song. The dancing robots' performance style must match the music style. In recent years, researchers have applied neural networks (NNs) to audio signal processing (ASP) (Jiang, 2020; Er et al., 2021). Over time, many variants of RNN have been developed and applied to ASP. This work optimizes the RNN to use for music style recognition. Then, an endpoint detector algorithm (EDA) based on short-term energy difference is proposed. The starting point of notes can be determined by looking for the peak of short-term energy difference. Then, two layers of judgment are designed to determine the endpoint and reduce the dependence on the threshold (Chakraborty et al., 2021; Feng et al., 2021).

**Figure 1:**
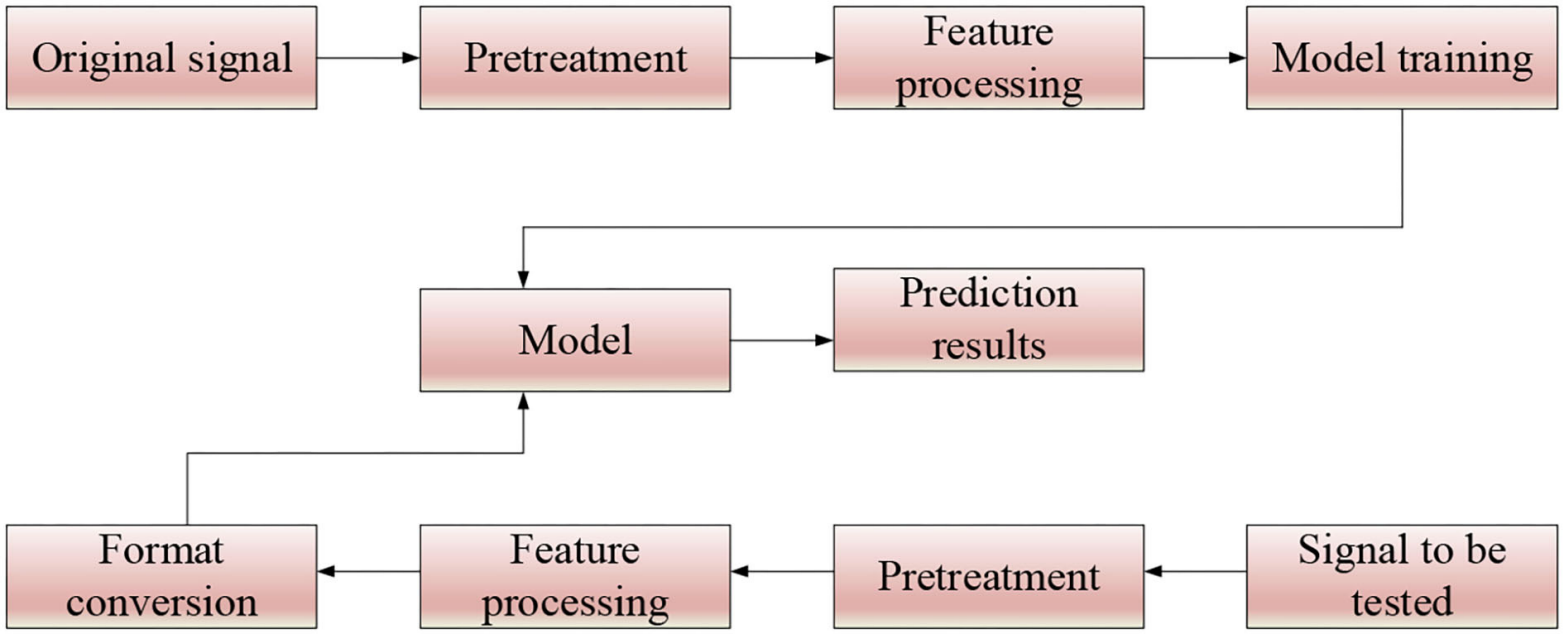
Framework of proposed EDA.

The principle of the proposed EDA is shown in Figure 1.

As in Figure 1, the music style recognition process is divided into two stages. The original signal is pre-processed in the training stage, and then the improved RNN is trained using the pre-processed data. In the testing stage, the audio to be tested is first pre-processed by simple data, and then the feature file is transformed (Mcauley et al., 2021; Wang et al., 2021).

**Figure 2:**
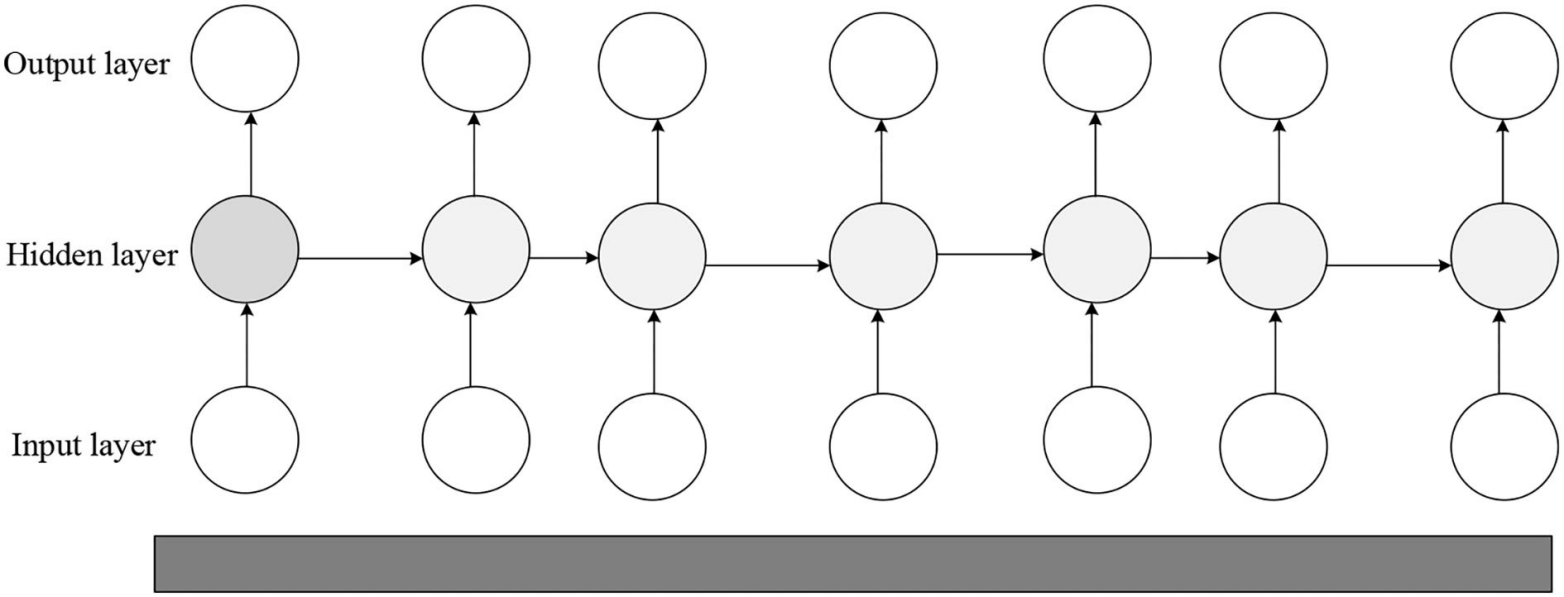
RNN structure.

DL is one of the main ways to lead to AI. DL is a branch of ML that essentially fits data to summarize the available laws. DL has successfully promoted science and technology and profoundly impacted big data analytics (BDA). A convolutional neural network (CNN) is one of the most important models in DL and lends well to image processing (IP). Combined with other technologies, CNN can be applied to many different fields. LeNet was the first real CNN proposed in 1998. This network has been widely used to recognize the handwritten font of Bank of America check, which has achieved good results. RNN is another commonly used DL structure and has a memory function. It is suitable for solving continuous sequence problems and is good at learning rules between samples with certain sequential significance. Unlike CNN, RNN is generally used in production and prediction, such as in Google Translate and some speech recognition applications (Mirza and Siddiqi, 2020; Wu, 2021). RNN is widely used in language models and text production, image description, video tagging, keyword extraction, and stock analysis. Meanwhile, RNN has a feedback structure. Its output relates both to the current input's weight and to the previous network's input. The difference between RNN and the traditional NN is that RNN has the concept of timing, and the state of the next moment will be affected by the current state. Some researchers also call recurrent networks deep networks, whose depth can be shown in input, output, and time-depth (Hernandez-Olivan et al., 2021; Parmiggiani et al., 2021). The RNN structure is given in Figure 2.

Equation (1) can calculate the hidden state *h*^*t*^ in the forward propagation of RNN:


(1) $$\begin{array}{l} h^{t}=\tanh\left(Ux^{t}+Wh^{t-1}+b\right) \end{array}$$

The network output at a specific moment can be calculated by Equation (2)


(2) $$\begin{array}{l} o^{t}=Vh^{t}+c \end{array}$$

The prediction output can be counted by Equation (3):


(3) $$\begin{array}{l} a^{t}=\mathrm{softmax}\left(o^{t}\right)=\mathrm{softmax}\left(Vh^{t}+c\right) \end{array}$$

In Equations (1)–(3), *x*^(*t*)^ represents the input of training samples when the sequence index number is *t*. *h*^(*t*)^ denotes the hidden state of the model when the sequence index number is *t*. *h*^(*t*)^ is jointly decided by *h*^(*t*)^ and *x*^(*t*)^. *o*^(*t*)^ signifies the output of the model when the sequence index number is *t*. *o*^(*t*)^ is only determined by the current hidden state *h*^(*t*)^. *L*^(t)^ refers to the model loss function when the sequence index number is *t*. signifies the true output of the training sample sequence when the sequence index number is *t*. Three matrices () are the model's linear relationship parameters, shared within RNN. The parameter sharing mechanism reflects the “recurrent feedback” of the RNN model.

For RNN, since there is a loss function at each position of the sequence, the final loss *L* can be explained by Equation (4):


(4) $$\begin{array}{l} L=\overset{\Gamma }{\underset{t=1}{\sum }}L^{\left(t\right)} \end{array}$$

The parameter gradient calculation reads:


(5) $$\begin{array}{l} \frac{\partial L}{\partial c}=\overset{\Gamma }{\underset{t=1}{\sum }}\frac{\partial L^{\left(t\right)}}{\partial c}=\overset{\Gamma }{\underset{t=1}{\sum }}{ŷ}^{\left(t\right)}-y^{t} \end{array}$$

(6) $$\begin{array}{l} \frac{\partial L}{\partial V}=\overset{\Gamma }{\underset{t=1}{\sum }}\frac{\partial L^{\left(t\right)}}{\partial V}=\overset{\Gamma }{\underset{t=1}{\sum }}\left({ŷ}^{\left(t\right)}-y^{t}\right){\left(h^{\left(t\right)}\right)}^{T} \end{array}$$

**Figure 3:**
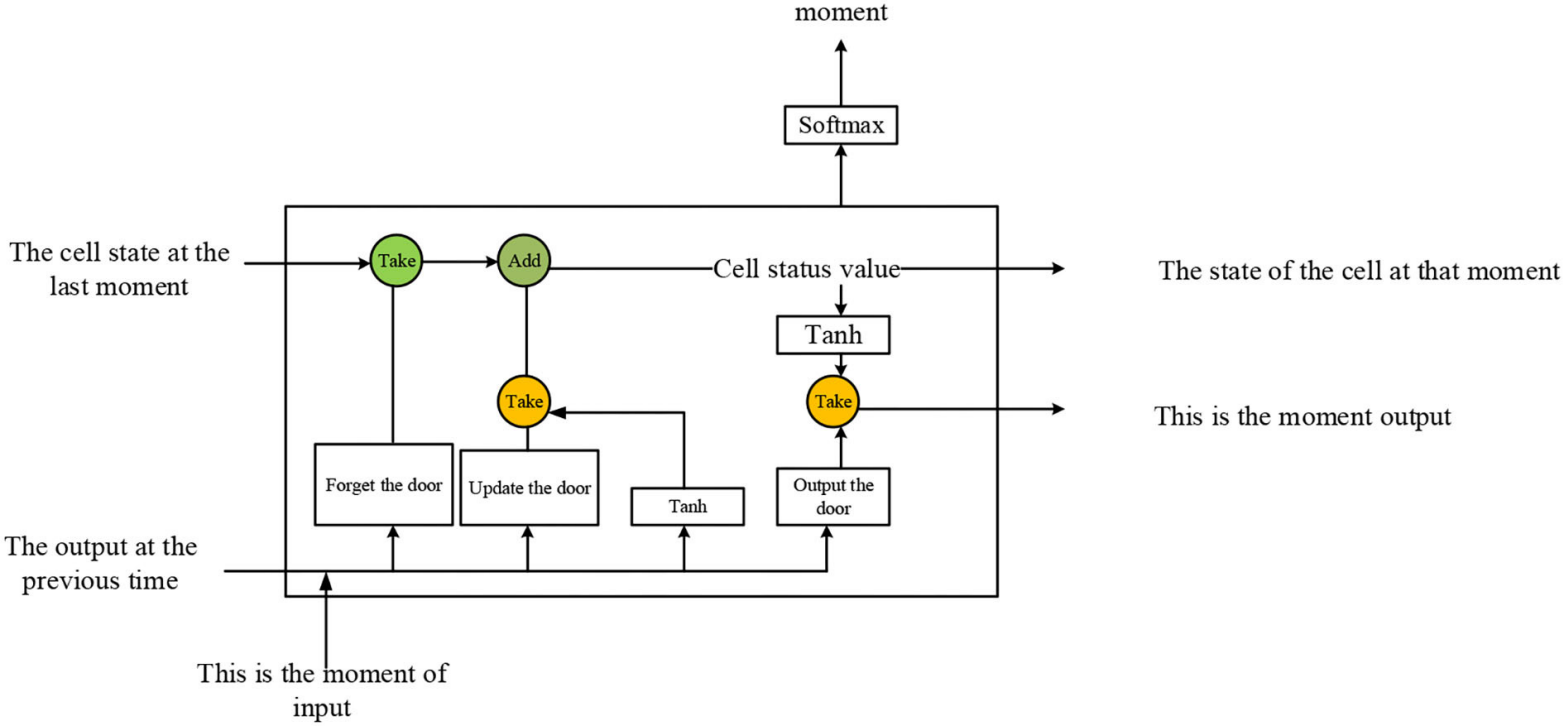
Original structure of the LSTM network.

RNN adds the concept of timing; thus, different input layers can be set according to the time node. Data can be entered in multiple ways. The number of hidden layers in the middle is the same as the number of time nodes, and the number of neurons per layer and independent variables are the same. The disadvantage of RNN is that it cannot solve the problem of long-term dependence, and there is a phenomenon of network gradient dissipation and explosion. Against the defect of RNN, the LSTM NN is proposed (Liu et al., 2021; Alfaro-Contreras et al., 2022), as drawn in Figure 3.

In LSTM, the forget and input gates are expressed by Equations (7) and (8), respectively. The short-term and long-term cell states are counted by Equations (9) and (10). The output gate is exhibited by Equation (11).


(7) $$\begin{array}{l} f_{t}=\sigma \left(W_{f}\cdot \left[h_{t-1},x_{t}\right]+b_{f}\right) \end{array}$$

(8) $$\begin{array}{l} i_{t}=\sigma \left(W_{i}\cdot \left[h_{t-1},x_{t}\right]+b_{i}\right) \end{array}$$

(9) $$\begin{array}{l} {\tilde{C}}_{t}=\tanh\left(W_{C}\cdot \left[h_{t-1},x_{t}\right]+b_{C}\right) \end{array}$$

(10) $$\begin{array}{l} C_{t}=f_{t}\cdot C_{t-1}+i_{t}\cdot {\tilde{C}}_{t} \end{array}$$

(11) $$\begin{array}{l} o_{t}=\sigma \left(W_{o}\cdot \left[h_{t-1},x_{t}\right]+b_{o}\right) \end{array}$$

LSTM and gated recurrent unit (GRU) are the first proposed two RNN variants. However, the practical applications find that the Sigmoid and Tanh functions in LSTM and GRU will lead to gradient attenuation and significantly impact the input of long-term sequences. To solve the above problems, this section introduces an IndRNN (Tan et al., 2021; Wagener et al., 2021). The hidden state in traditional RNN is the input of the next state and is updated by Equation (12):


(12) $$\begin{array}{l} h_{t}=\sigma \left(WX_{t}+Uh_{t-1}+b\right) \end{array}$$

In Equation (12), *h*_*t*_ is the hidden state at time *t*. *h*_*t*−1_ represents the hidden state at the previous moment. *U* is the weight of different stages.

According to relevant literature, the multiplication operation of recurrent weight causes gradient explosion or attenuation. The IndRNN adopts a new and independent RNN as the basic classification model. Unlike traditional RNN, IndRNN employs a different state update mechanism (Li and Zheng, 2021; Xu et al., 2021). Its recurrent input is processed by Hadamard product, as in Equation (13):


(13) $$\begin{array}{l} h_{t}=\sigma \left(WX_{t}+u⊙h_{t-1}+b\right) \end{array}$$

In Equation (13), *u* is a recurrent weight. Its mathematical form is a vector. ⊙ is a Hadamard product operation. The principle is to multiply the corresponding elements of the two matrices before and after the symbol.

At moment *t*, each neuron only accepts the input at this moment and its own hidden state as input information at moment *t* − 1. The hidden state of the *n*th neuron is described by Equation (14):


(14) $$\begin{array}{l} h_{n,t}=\sigma \left(W_{n}X_{t}+u_{n}h_{n,t-1}+b_{n}\right) \end{array}$$

In Equation (14), *W*_*n*_, *u*_*n*_ is the *n*th line of input weight and recurrent weight. *W* and *u* input the spatial and temporal features, respectively.

**Figure 4:**
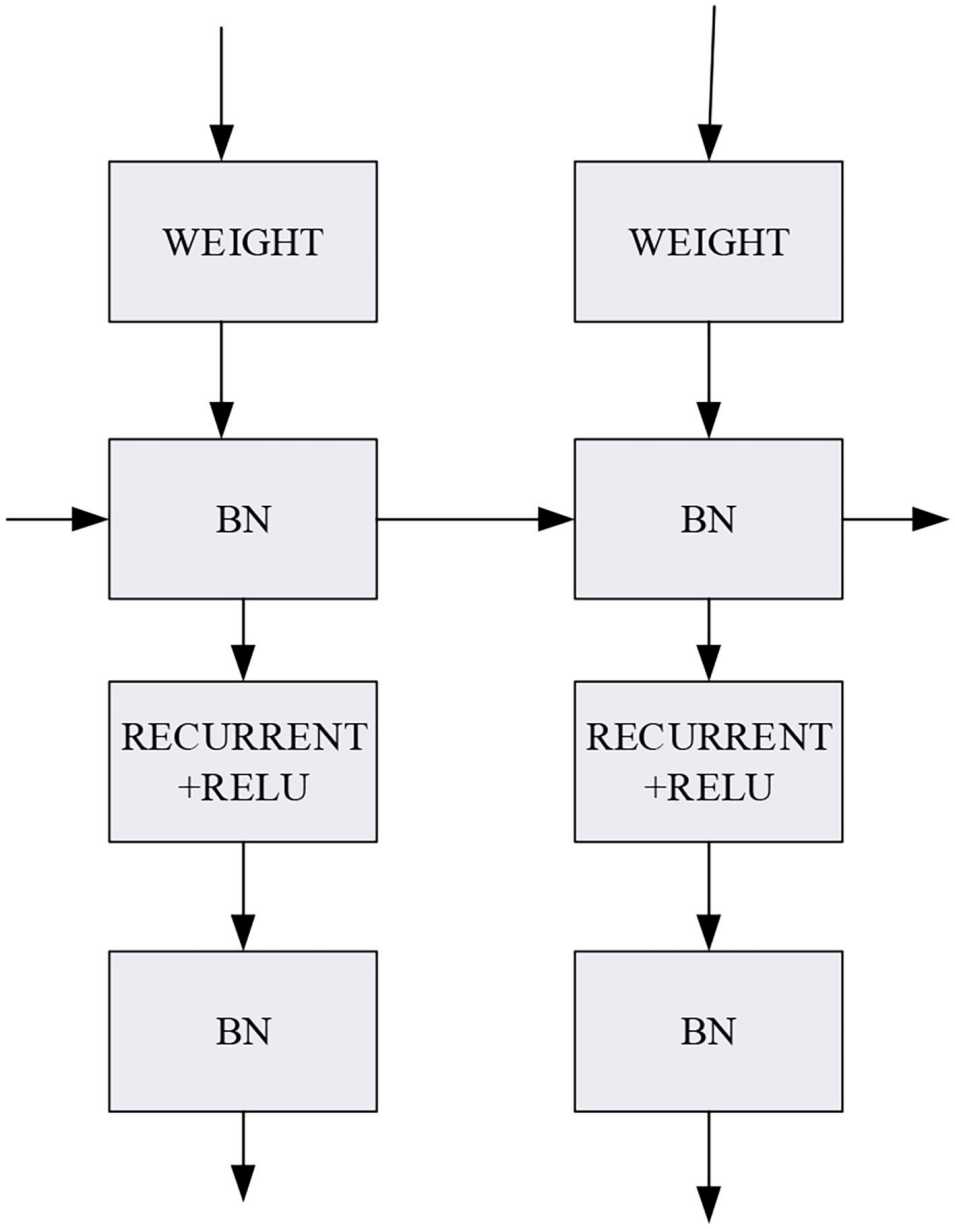
IndRNN.

The basic architecture of the IndRNN is depicted in Figure 4. Activation function (AF) chooses the rectified linear unit (ReLU). The IndRNN processes the input weight using the Recurrent + ReLU structure.

The IndRNN back propagates the gradient in each layer according to the temporal features. For the *n*th neuron *h*_*n,t*_, if the optimization goal of *T*-time is *J*, then at *t*-time, the gradient reverse propagation process is described by Equations (15)–(18):


(15) $$\begin{array}{l} \frac{\partial J_{n}}{\partial h_{n,t}}=\frac{\partial J_{n}}{\partial h_{n,T}}\frac{\partial h_{n,T}}{\partial h_{n,t}} \end{array}$$

(16) $$\begin{array}{l} \frac{\partial J_{n}}{\partial h_{n,t}}=\frac{\partial J_{n}}{\partial h_{n,T}}\underset{k=t}{\prod }\frac{\partial h_{n,k+1}}{\partial h_{n,k}} \end{array}$$

(17) $$\begin{array}{l} \frac{\partial J_{n}}{\partial h_{n,t}}=\frac{\partial J_{n}}{\partial h_{n,T}}\overset{T-1}{\underset{k=t}{\prod }}\sigma_{n,k+1}^{\prime }u_{n} \end{array}$$

(18) $$\begin{array}{l} \frac{\partial J_{n}}{\partial h_{n,t}}=\frac{\partial J_{n}}{\partial h_{n,T}}u_{n}^{T-t}\overset{T-1}{\underset{k=t}{\prod }}\sigma_{n,k+1}^{\prime } \end{array}$$

$\sigma_{n,k+1}^{\prime }$ is the AF in Equation (18).

According to Equation (18), the gradient of the IndRNN directly depends on *u_n_* index, while the traditional RNN gradient is calculated by Equation (19):


(19) $$\begin{array}{l} \frac{\partial J_{n,T}}{\partial h_{n,t}}=\overset{T}{\underset{t=0}{\sum }}\frac{\partial J_{n,T}}{\partial {ŷ}_{n,T}}*\frac{\partial {ŷ}_{n,T}}{\partial h_{n,T}}\overset{T}{\underset{j=t+1}{\prod }}\frac{\partial h_{n,j}}{\partial h_{n,j-1}} \end{array}$$

The traditional RNN determines the gradient by the Jacobian matrix. A slight change in the matrix might cause great fluctuation in the final output. In summary, compared with traditional RNN, IndRNN has many advantages in long-term sequence tasks. First, IndRNN can avoid gradient disappearance and explosion more effectively. Second, IndRNN can process long-term sequences better. Finally, IndRNN has a better explanation (Mussoi, 2021; Shalini et al., 2021; Xu, 2022).

**Figure 5:**
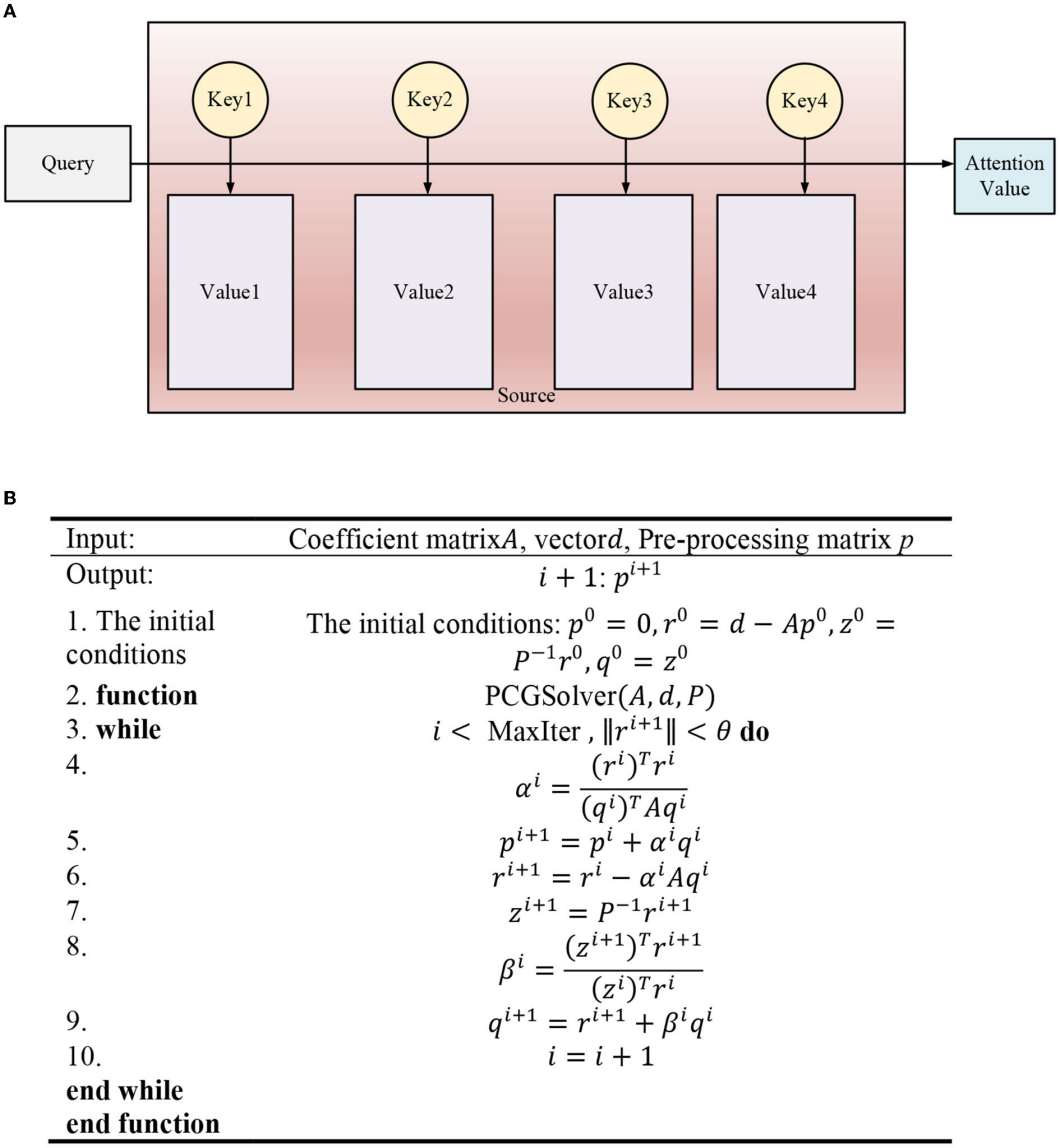
Thought of AM. **(A)** Network structure, **(B)** Algorithm pseudocode.

Visual AM is a unique signal processing mechanism of the human brain. Human beings can choose areas of focus by observing global pictures (Mussoi, 2021; Zainab and Majid, 2021). Thereby, they devote more resources to the focus area than ordinary areas to obtain more detailed features while suppressing useless information. The essence of the AM is illustrated in Figure 5.

**Figure 6:**
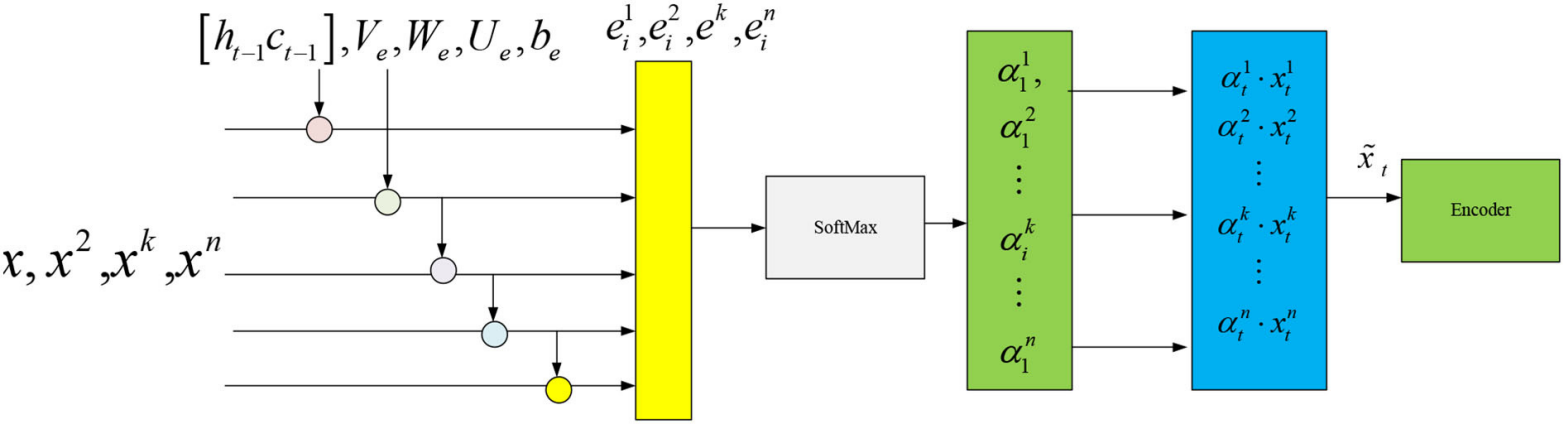
Input AM module calculation process.

The input data of the input AM module is *X* = (*x*^1^, *x*^2^, ⋯  , *x*^*k*^ ⋯  , *x*^*n*^), representing *n* environmental variable sequences. ${\text{x}}^{k}=\left(x_{1}^{k},x_{2}^{k},\cdots \;,x_{t}^{k},\cdots x_{T}^{k}\right)$ denotes the *k*th environment variable sequence, and the time window size is *T*. The hidden state ***h***_*t*−1_ of the previous time corresponding to the input of the LSTM unit and cell state ***C***_*t*−1_-extracted environmental parameter weights are introduced into the input AM module. The calculation process is shown in Figure 6, where $e_{t}^{k}$ and $\alpha_{t}^{k}$ are calculated by Equations (20) and (21):


(20) $$\begin{array}{l} e_{t}^{k}=V_{e}\tanh\left(W_{e}\left[h_{t-1}:c_{t-1}\right]\right)+U_{e}x^{k}+b_{e}) \end{array}$$

(21) $$\begin{array}{l} \alpha_{t}^{k}=\frac{\exp\left(e_{t}^{k}\right)}{\sum_{i=1}^{n}\exp\left(e_{t}^{i}\right)} \end{array}$$

In Equations (20) and (21), $e_{t}^{k}$ represents the weight of the *k*th environmental parameter at time *t*. $\alpha_{t}^{k}$ k means the value of $e_{t}^{k}$ normalized by softmax function. ***V***_*e*_, ***W***_*e*_, ***U***_*e*_, and ***b***_*e*_ are the parameter to be trained.

Equation (22) calculates the environmental parameter's vector ${\tilde{\text{x}}}_{t}$ weighted by $\alpha_{t}^{k}$ at time *t*:


(22) $$\begin{array}{l} {\tilde{\text{x}}}_{t}=\left(\alpha_{t}^{1}x_{t}^{1},\alpha_{t}^{2}x_{t}^{2},\cdots \;,\alpha_{t}^{k}x_{t}^{k},\cdots \alpha_{t}^{n}x_{t}^{n}\right) \end{array}$$

**Figure 7:**
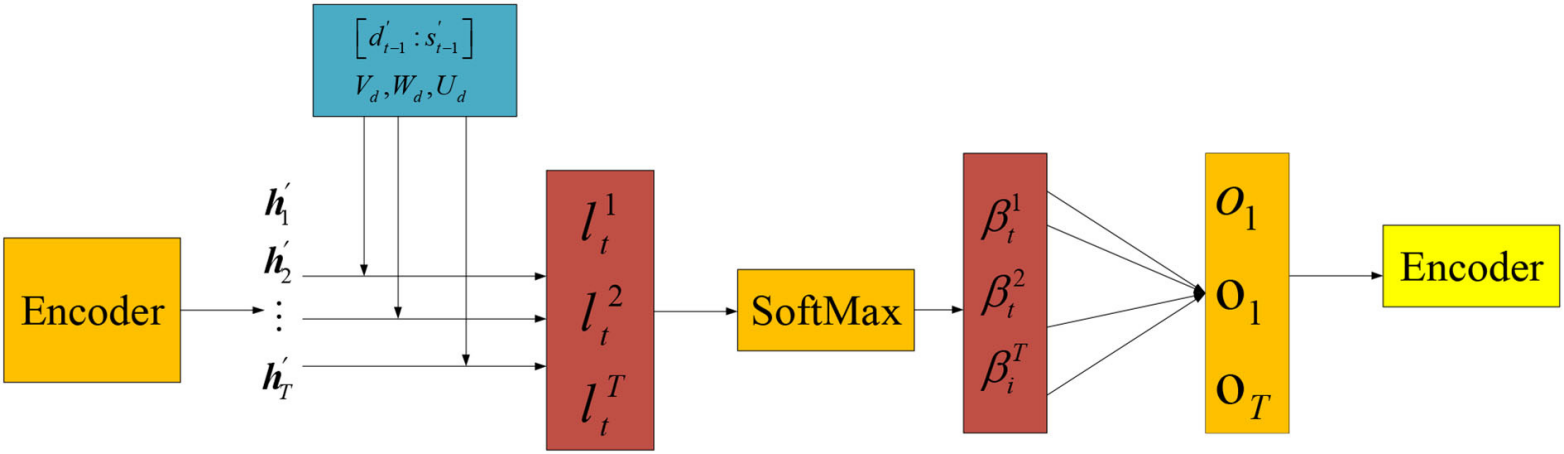
Temporal AM.

The temporal AM extracts the importance of environmental variables at different times, and its calculation process is shown in Figure 7.

The input data are the output of the encoder module. That is the hidden state $h_{i}^{\prime }$ of the weighted value ${\tilde{\text{x}}}_{t}$ at time *i* of the environmental data sequence after passing through the LSTM unit. The temporal attention weight is calculated by Equations (23) and (24):


(23) $$\begin{array}{l} l_{t}^{i}=V_{d}\tanh\left(W_{d}\left[d_{t-1}^{\prime }:s_{t-1}^{\prime }\right]\right)\text{ + }U_{d}h_{i}^{\prime }) \end{array}$$

(24) $$\begin{array}{l} \beta_{t}^{i}=\frac{\exp\left(l_{t}^{i}\right)}{\sum_{j=1}^{T}\exp\left(l_{t}^{j}\right)} \end{array}$$

In Equations (23) and (24), 1 < *i* ⩽ *T, T* is the size of the time window. $d_{t-1}^{\prime }$ and $S_{t-1}^{\prime }$ are the hidden state and cell state at (*t* − 1) time. ***V***_*d*_, ***W***_*d*_, and ***U***_*d*_ are the parameter to be trained. $\beta_{t}^{i}$ represents the normalized weight of the *i*th hidden state.

Output ***O***_*t*_ of temporal AM module is calculated by Equation (25):


(25) $$\begin{array}{l} {\text{O}}_{t}=\overset{T}{\underset{i=1}{\sum }}\beta_{t}^{i}h_{i}^{\prime } \end{array}$$

The decoder predicts the beat at time *T* + 1 combined with the fully connected layer. ***Y*** = (*y*_1_, *y*_2_, ⋯  , *y*_*t*_, ⋯  , *y*_*T*_) represents the number of beats at each time in the *T* time window. The specific process is shown in Equations (26) and (27).


(26) $$\begin{array}{l} {ỹ}_{t}=\text{W}\left[y_{t}:{\text{O}}_{t}\right]+\tilde{b} \end{array}$$

(27) $$\begin{array}{l} d_{t+1}^{\prime }=f\left(d_{t}^{\prime },{\tilde{y}}_{t}\right) \end{array}$$

In Equations (26) and (27), ***O***_*t*_ is the output of the temporal AM module. ỹ_*t*_ is a linear transformation of *y*_*t*_. $d_{t}^{\prime }$ represents the hidden state of the decoder at time *t*. $d_{t+1}^{\prime }$ denotes the hidden state of the decoder at the time (*t* + 1). Function *f* is an LSTM computing unit. ***W*** and $\tilde{b}$ are parameters to be trained.

### Design of interactive humanoid robot system

Interactive robots are dancing robots expressing artistic forms, such as action and language, using physical movements. The dancing robot can recognize the music style and beat for given music and display the right music style and beats. Generally, music style has two targets, namely, beat recognition and action performance. Beat recognition results can be expressed from the actions of interactive robots. Pose estimation is to extract dance movements to form a dance action database from many dance videos. Finally, the interactive robot performs specific actions according to the recognized beat. Dance movement extraction can generate intelligent choreography based on pose estimation technology. By comparison, pose estimation is a basic computer vision technology, the estimation of the human posture or the key points of the human body. The dance action library comprises various stylish dances, divided into ten categories. The dance library is mainly divided into two parts, aiming at robots and non-professionals. The design framework of dancing robots is divided into three parts. The focus is on the design of the dance library, which is divided into three steps. First, the image is detected from the video frame. Second, 2D keypoint information is detected from the image. Third, the music style recognition system converts the 2D keypoint information into 3D information. Fourth, the 3D information is transformed into joint angle information recognizable by the robot motion model. Ultimately, the dance action library is enriched according to the actions obtained from different styles of dance videos. Dance pose estimation and the dance action classification modules in the HCI system are the key to background recognition. Accuracy and response time can evaluate the dance movements and test the feasibility of HCI systems based on dance education and action analysis and recognition.

The deep LSTM network architecture reported here contains a four-layer network structure. The first layer is the input, with 13 neuron nodes. The neurons in the middle two hidden layers are 128 and 32, respectively. The last layer is the output containing ten nodes corresponding to ten music styles.

### The dataset and the environment configuration of the experiment

The music style library used in this experiment is GTZAN. It is the western music style library used by Tzanetakis in his paper published in 2002, including ten music styles, namely, blues, classical music, country music, disco, hip-hop, jazz, metal, pop music, reggae, and rock, with 100 clips in each style, totalling 1,000 music clips. Each segment is a mono 16-bit *wav* file with a length of 30 s and a sampling rate of 22.05 kHz. Of these, 50 clips are selected from each style for training, 25 clips for verification, and 25 clips for testing.

## Analysis and discussion of experimental results

### Experimental results of model parameter

**Figure 8:**
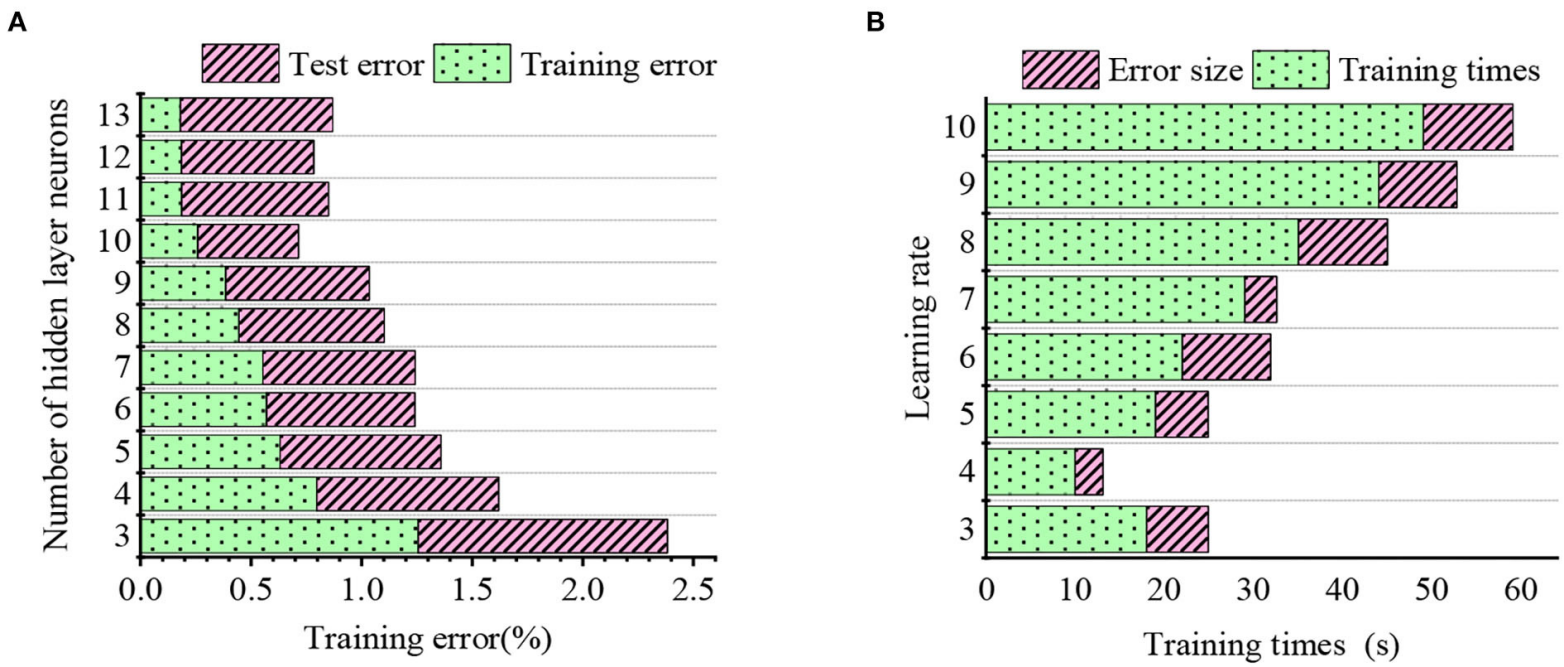
Relationship between the hidden layer neurons, the learning rate, and model error. **(A)** Indicates the relationship between the hidden layer neurons and error. **(B)** Shows the relationship between the learning rate and model error.

The relationship between the hidden layer neurons, the learning rate, and model error is outlined in Figure 8.

Overall, the training error decreases when the hidden layer neurons increase from 3 to 13. In particular, when the hidden layer neurons increase from 10, 11, to 12, the training error increases first and then decreases. In conclusion, the optimal hidden layer nodes are 11. On the other hand, the learning rate directly affects the model learning and training efficiencies. Concretely, the prediction error fluctuates greatly given a large learning rate, and the model converges fast. By comparison, a low learning rate means some uncertainties and slow convergence. Finally, the optimal learning rate is determined as 0.01 by observing errors and the number of training.

### Functional test of the algorithm

**Figure 9:**
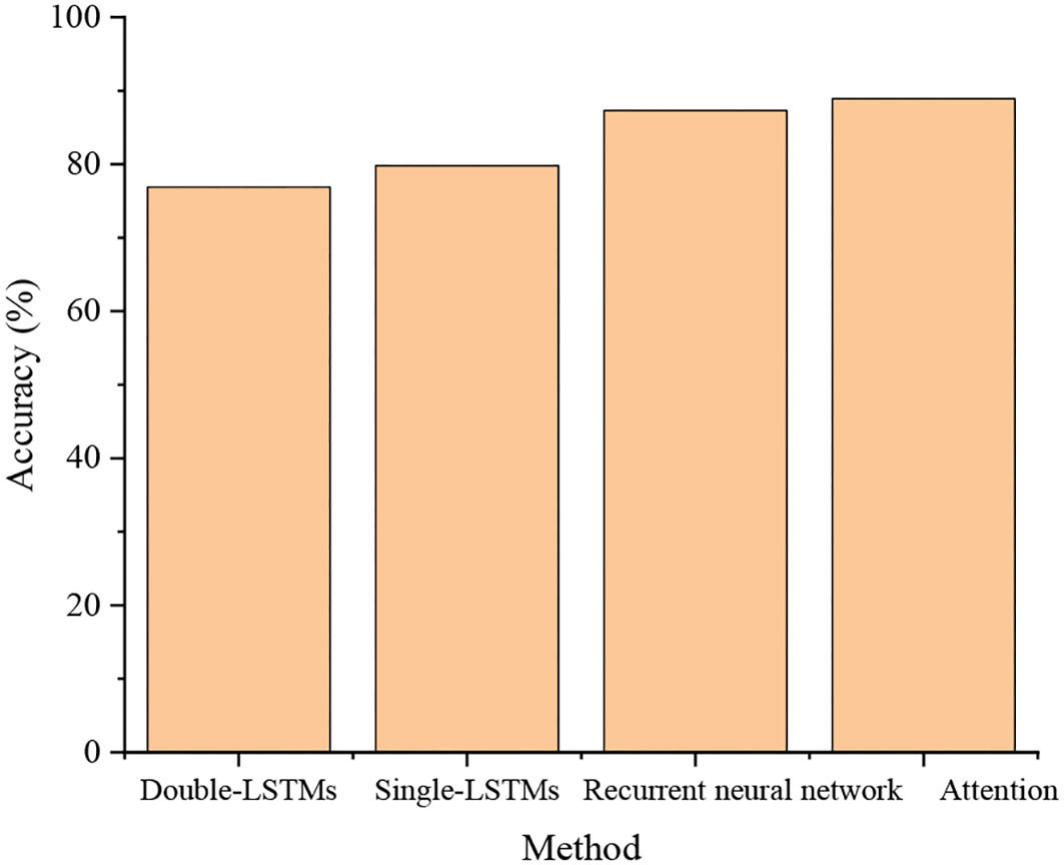
Model comparison results.

Figure 9 compares the music style and beats recognition accuracy of single LSTM, bi-directional LSTM, IndRNN, and AM-IndRNN.

Comparing the loss and prediction accuracy reveals that multi-parallel models have higher prediction accuracy than single ones. The recognition accuracy of the multi-parallel model reaches 79.8%, higher by 43.8% than the single LSTM model, and the model loss is only 6.85%. Overall, the recognition accuracy and loss of the AM-IndRNN reported here are optimal, reaching 88.9% and 7.48%, respectively. Therefore, the optimized LSTM has higher recognition accuracy and is more applicable for recognizing music styles and beats.

**Figure 10:**
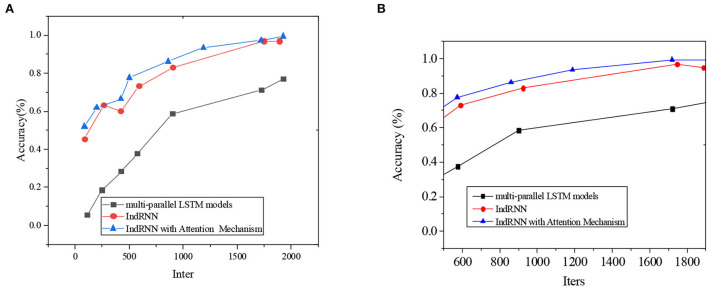
Comparison of multi-parallel LSTM models, IndRNN, and AM-IndRNN. **(A)** the first test; **(B)** the second test.

The result of note prediction accuracy is plotted in Figure 10.

In Figure 10, the abscissa means the number of iterations, and the ordinate denotes the note prediction accuracy. Apparently, note prediction accuracy increases with training iterations. Meanwhile, the accuracy of the proposed AM-IndRNN is always higher than that of multi-parallel LSTM models and IndRNN.

The experimental results show that compared with the model proposed by Soufineyestani et al. (2021), the AM-IndRNN reported here has a higher recognition rate on the GTZAN dataset. The experimental results of this paper are compelling. They can well complete the classification of music styles on the GTZAN dataset.

The advantage of the AM-IndRNN reported here is that the recognition accuracy and loss rate are optimal, reaching 88.9% and 0.0748, respectively. Compared with the non-optimized LSTM model, the optimized LSTM model has higher recognition accuracy.

The disadvantage of the AM-IndRNN reported here is that this work limits the research object to single music tone recognition. There are different musical instruments in different countries and nationalities. With the deepening of research, the recognition task may no longer be limited to specific musical instruments or single musical instrument performance. With the continuous expansion of instrumental music, the identification and discrimination work can eventually develop into the performance identification of multiple groups of musical instruments and even the music performance identification with vocal music elements.

### Algorithm comparison

**Figure 11:**
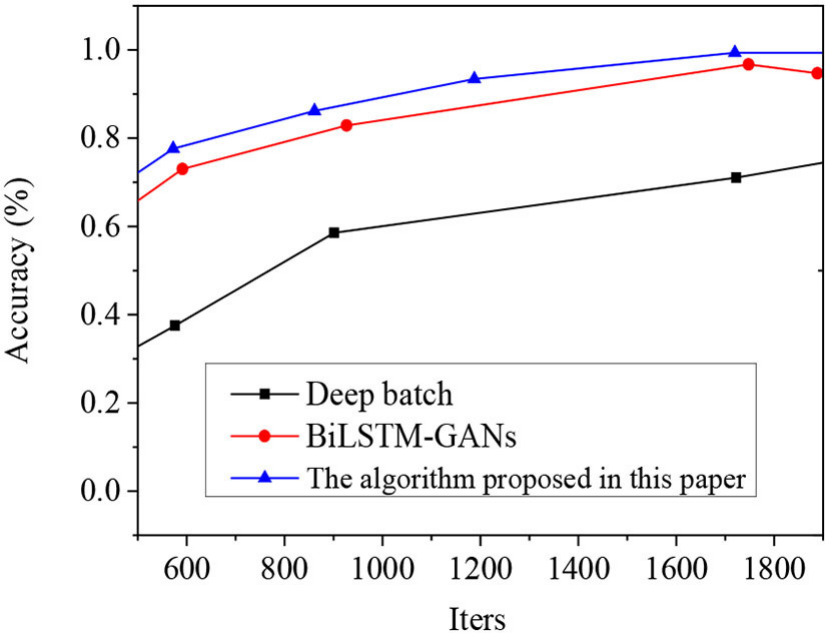
Comparison of the deep Bach model, BiSTM-GAN model, and the proposed AM-IndRNN model.

Under the same experimental conditions, the proposed AM-IndRNN model, deep Bach model, and BiLSTM- Generative adversarial network (GAN) model's note prediction accuracies are compared in Figure 11.

According to Figure 11, when the network iteration = 600, the music note prediction accuracy of the proposed AM-IndRNN, BiLSTM-GAN, and deep Bach model is 73, 65, and 33%, respectively. When the network iteration = 1,400, the accuracy of the above three models is 92, 85, and 53%, respectively. Therefore, with the increase in network iteration, the model's accuracy in predicting notes gradually increases. Meanwhile, the accuracy of the proposed AM-IndRNN to predict notes is always higher than deep Bach and BiLSTM-GAN models.

## Conclusion

With the popularity of internet technology and multimedia equipment, online digital music has increased exponentially. Thus, it becomes extremely challenging to manually manage and classify massive numbers of online musical works. At the same time, users' needs for timely and accurate music information retrieval have become imminent. This requires the design of an accurate and effective music style and beats recognition and classification system to manage online music databases. The traditional music style classification methods need a priori knowledge, with complex feature extraction and fewer representative features. In particular, the DL classification model can be used for automatic music style classification. This work focuses on music style classification from audio feature extraction, classifier training, and music style prediction. As a result, a complete automatic music style recognition system is implemented. To do so, the LSTM model is selected over CNN. The sample music is divided into ten different styles. Meanwhile, a hierarchical classification is adopted to improve the classification accuracy further. Specifically, music is classified into strong and weak categories by the LSTM classifier and then divided into multiple subcategories. This new multi-stage classification method is used to classify different music styles. Experiments show that hierarchical multi-step can improve classification accuracy to a certain extent.

However, there are still some deficiencies. The music style recognition system reported here recognizes single notes, but multiple notes generally appear continuously in real music. Hence, future research work will continue to study the problem of multi-tone pitch recognition. In addition, due to the limitation of research time, the number of samples is small, and the experimental samples will continue to be expanded in future research.

## Data availability statement

The raw data supporting the conclusions of this article will be made available by the authors, without undue reservation.

## Author contributions

The author confirms being the sole contributor of this work and has approved it for publication.

## Funding

This work was supported by the Research on Digital Inheritance and Protection of Intangible Cultural Heritage Music of Ethnic Minorities in Liaoning Province, 2019, Liaoning social science planning fund project (No. L19BMZ007).

## Conflict of interest

The author declares that the research was conducted in the absence of any commercial or financial relationships that could be construed as a potential conflict of interest.

## Publisher's note

All claims expressed in this article are solely those of the authors and do not necessarily represent those of their affiliated organizations, or those of the publisher, the editors and the reviewers. Any product that may be evaluated in this article, or claim that may be made by its manufacturer, is not guaranteed or endorsed by the publisher.
